# Staphylococcus lugdunensis Urinary Tract Infection With Associated Neutropenic Fever

**DOI:** 10.7759/cureus.21432

**Published:** 2022-01-19

**Authors:** Rajanish Bobde, Joseph I Berger, Urma Jalil, Garine Kalaydjian

**Affiliations:** 1 Infectious Disease, St. John's Riverside Hospital, Yonkers, USA; 2 Internal Medicine, St. John's Riverside Hospital, Yonkers, USA; 3 Internal Medicine, Lake Erie College of Osteopathic Medicine, Greensburg, USA

**Keywords:** thrombocytopenia, chemotherapy, neutropenia, neutropenic fever, staphylococcus lugdunensis, urinary tract infection

## Abstract

We present a 62-year-old woman with a history of uterine cancer status post-total abdominal hysterectomy and bilateral salpingo-oophorectomy (TAH-BSO) on paclitaxel, who presented to the emergency department febrile at 101.7 Fahrenheit and complaining of fatigue and urinary incontinence. Laboratory testing revealed neutropenia and urinalysis showed elevated bacteria with minimal white blood cells, and negative leukocyte and negative nitrites. Urine cultures ultimately showed *Staphylococcus lugdunensis* with negative blood cultures.

*S. lugdunensis* is a less frequently speciated Staphylococcus and has been increasingly found due to advances in identification using matrix-assisted laser desorption ionization time of flight mass spectrometry (MALDI-TOF MS). *S. lugdunensis* are Gram-positive cocci, nonsporulating, nonmotile, facultatively anaerobic, catalase-positive, coagulase-negative, oxidase-negative, delta-hemolytic organism. Traditionally, it is seen in skin and soft-tissue infections, as well as vascular infections, however, has minimal occurrences in urinary tract infections.

The risk of infection is increased in immunocompromised states and empiric treatment is warranted while waiting for definitive results. Our patient was started on cefepime, valacyclovir, fluconazole, and a single dose of vancomycin while in the emergency department. Worsening thrombocytopenia during her antibiotic course necessitated the re-evaluation of antibiotic agents which can cause thrombocytopenia. Subsequently, due to the patient’s improved clinical status, and low risk of severe outcome, fluconazole and valacyclovir were discontinued, and cefepime was changed to ceftriaxone.

## Introduction

*Staphylococcus lugdunensis* was discovered in France in 1988 and has been increasingly isolated over the past decades [1]. Based on 11 clinical strains, *S. lugdunensis* have been categorized as Gram-positive cocci, nonsporulating, nonmotile, facultatively anaerobic, catalase-positive, coagulase-negative, oxidase-negative, delta-hemolytic, and susceptible to novobiocin [1]. Since that time, *S. lugdunensis* has been found over the entire surface of the human skin, but more commonly in the inguinal folds and lower half of the body [2-3]. Studies showed that the most common clinical diagnoses were skin and soft-tissue infections (55.4%) and vascular-associated infections (17.4%) [4]. There are very few occurrences of this bacteria causing urinary tract infections (UTIs). [5] One large-scale study demonstrated that only 6% of urine specimens (31 of a total of 4,652) found *S. lugdunensis*, with the greatest prevalence in women aged 65 or older [6]. Typically, infections were thought to sprout from trauma or immunosuppression, however, they have been found in healthy individuals as well [5]. Notably, there has been an increased incidence of infections with *S. lugdunensis* since 2014, likely due to the introduction of the matrix-assisted laser desorption ionization time-of-flight mass spectrometry which has offered a revolutionary technique for the identification of bacteria [7].

## Case presentation

Our patient is a 62-year-old female with a past medical history of uterine cancer status post-total abdominal hysterectomy-bilateral salpingo-oophorectomy (TAH-BSO), currently on paclitaxel (last therapy 10 days prior to admission), who presented to the hospital complaining of fatigue, fever, and urinary incontinence. Subsequent laboratory testing showed her to be neutropenic. Temperature max (Tmax) recorded at home was 101.7 degrees Fahrenheit, after which the patient took acetaminophen and presented to the hospital. Vitals, labs, urinalysis, urine culture, and blood cultures were ordered. Urinalysis showed elevated bacteria and minimal amounts of WBCs with no leukocyte esterase and no nitrates (Table 1). Cefepime, valaciclovir, fluconazole, and a single dose of vancomycin were started empirically in the emergency room. Following her 2nd day, Tmax peaked, and subsequently down-trended until she became afebrile on day 3. Filgrastim was administered on the 3rd hospital day and resulted in a transient increase in WBCs, with no effect on platelet count which had continued to decrease. Her urine culture on day 5 reported *S. lugdunensis* sensitive to ceftriaxone as well as minimal amounts of *Enterococcus faecalis*. Blood cultures returned negative. Due to our patient’s low risk for complication as determined by her Multinational Association for Supportive Care in Cancer (MASCC) score of 24, and Clinical Index of Stable Febrile Neutropenia (CISNE) score of 1, cefepime, valaciclovir, and fluconazole were discontinued due to their potential to decrease platelets, and ceftriaxone was started instead and continued for 4 more days [8-10]. WBCs dropped again between days 5 through 7, and platelets decreased between days 7 and 8. A second dose of filgrastim was administered on day 7 resulting in an up-trending WBC on day 8 and an up-trending platelet count on day 9. WBCs declined on day 9, however, the absolute neutrophil count (ANC) remained in the normal range. Following this, the patient continued to recover and was discharged off antibiotics.

**Table 1:** Vitals and Laboratory Values °F: degrees Fahrenheit; bpm: beats per minute; mmHg: millimeters of mercury; SpO_2_: peripheral capillary oxygen saturation measured by pulse oximetry; K/mm^3^: thousand per cubic millimeter; gm/dL: grams per deciliter; % L/L: percentage liter of cells per liter of blood; K/µL: thousand cells per microliter; WBC/µL:   Leucocytes per microliter; RBC/µL: red blood cells per microliter; Bact/µL: bacteria cells per microliter

Vitals & Laboratory Values											
Home Vitals			Day 1								
Temperature (°F)			101.7								
Vitals:			Day 1	Day 2	Day 3	Day 4	Day 5	Day 6	Day 7	Day 8	Day 9
	Temperature (°F)		99.6	101.9	98.9	98.4	98.2	98.6	98.2	98.5	98.2
	Pulse (bpm)		128	107	82	80	91	87	86	89	67
	Blood Pressure (mmHg)		128/64	111/50	100/60	114/56	125/70	121/57	106/55	110/67	109/66
	Oxygen Saturation (SpO_2_ on room air)		97%	97%	96%	96%	97%	95%	97%	98%	99%
Laboratory Values:			Day 1	Day 2	Day 3	Day 4	Day 5	Day 6	Day 7	Day 8	Day 9
	White Blood Cells (K/mm^3^)		0.4	0.7	0.9	2.0	2.7	1.9	1.6	4.9	3.5
	Absolute Neutrophil Count (K/mm^3^)		0.0	0.0	0.1	1.1	1.5	0.8	0.7	3.9	2.3
	Hemoglobin (gm/dL)		8.0	8.2	7.8	7.3	7.3	7.2	7.2	7.9	7.6
	Hematocrit (% L/L)		23.6	24.0	22.8	21.6	21.3	21.3	21.2	23.3	22.7
	Platelets (K/µL)		61,000	51,000	40,000	36,000	30,000	30,000	27,000	25,000	28,000
Urinalysis:											
	White Blood Cells (WBC/µL)		20								
	Red Blood Cells (RBC/µL)		49								
	Bacteria (Bact/µL)		2,189								
	Leukocyte Esterase		Negative								
	Nitrites		Negative								
Urine Culture:											
		*Staphylococcus lugdunensis* > 100,000 colony-forming units									
		*Enterococcus faecalis* 20,000-30,000 colony-forming units									
Blood Culture:											
		No growth after 5 days									

## Discussion

Febrile neutropenia is the occurrence of a fever in the setting of an ANC less than 1500. This increases the chance of infection, and possibly the severity of the infection [11]. Risk of disease course can be estimated by the CISNE and MASCC scores and is considered to be low. It is standard therapy to be started on empiric antibacterial, antiviral, and antifungal treatment to prevent opportunistic infections [12]. Our patient suffered from persistent neutropenia as well as thrombocytopenia in the setting of recent chemotherapy. The increased susceptibility to infection, combined with profound thrombocytopenia required careful monitoring of both her antibiotic regimen and hemodynamic status.

Our patient developed a UTI with *S. lugdunensis* likely secondary to her neutropenia and the bacteria’s predilection for the inguinal area [2]. *S. lugdunensis*, in comparison to other coagulase-negative Staph species, has the potential for increased virulence due to its delta-like toxin resembling the delta-toxin in *Staphylococcus aureus* as well as increased resistance via acquiring the blaZ gene conferring penicillinase production. The delta-toxin of *S. aureus* increases virulence by forming membrane-damaging pores which result in cell lysis [7,13]. To date, the presence of mecA gene, and thus methicillin resistance, among *S. lugdunensis*, is rare ranging from 0 to 8.3% [13]. Interestingly, compared to *Staphylococcus saprophyticus*, another coagulase-negative Staphylococcus (albeit with resistance to novobiocin) which also colonizes a similar area as *S. lugdunensis*, *S. lugdunensis* is more associated with patients who have solid organ tumors [7]. This raises the question of whether cancer-related immunosuppressive states permit *S. lugdunensis* infection whereas an *S. saprophyticus* infection might otherwise flourish. Additionally, unlike *S. saprophyticus*, which infrequently causes extra-genitourinary infection, *S. lugdunensis* has been implicated in skin and soft-tissue infections, respiratory infections, bacteremia, prosthetic joint infections, and peritonitis [6].

It is important to note that despite the pan-sensitivity of our patient’s organism, we deemed it medically prudent to change antibiotics, and discontinue antifungal and antiviral therapy due to the patient’s persistent thrombocytopenia and overall low risk for viral and fungal pathogens. Reports have found correlations between antibacterial, antifungal, and antiviral medications causing thrombocytopenia, including cefepime, fluconazole, and valaciclovir [8-10].

While currently, most strains of *S. lugdunensis* are pan-sensitive to antibiotic therapy, due to its unique virulence and correlation with patients who have solid organ tumors as well as the potential for extra-genitourinary manifestation, we posit that greater surveillance for this bacteria is warranted, especially when it is isolated in cultures with colony-forming units greater than 100,000 [13].

## Conclusions

*S. lugdunensis* is still relatively unrecognized as a urogenital pathogen and has not been found in sufficient quantity in many urine cultures as of date. Its presence in patients with solid organ tumors however must be considered. While it has been studied in the setting of skin and soft-tissue infections, further studies are required on its frequency of causing UTIs, as well as the potential for increasing virulence and resistance due to its unique genes.
